# Phytol/Phytanic Acid and Insulin Resistance: Potential Role of Phytanic Acid Proven by Docking Simulation and Modulation of Biochemical Alterations

**DOI:** 10.1371/journal.pone.0045638

**Published:** 2013-01-02

**Authors:** Mohamed M. Elmazar, Hanan S. El-Abhar, Mona F. Schaalan, Nahla A. Farag

**Affiliations:** 1 British Universityin Cairo (BUE), Cairo, Egypt; 2 Department of Pharmacology & Toxicology, Faculty of Pharmacy, Cairo University, Cairo, Egypt; 3 Department of Biochemistry, Faculty of Pharmacy, Misr International University (MIU), Cairo, Egypt; 4 Department of Pharmaceutical Medicinal Chemistry, Faculty of Pharmacy, Misr International University (MIU), Cairo, Egypt; University of Texas Health Science Center at San Antonio, United States of America

## Abstract

Since activation of PPARγ is the main target for the antidiabetic effect of TZDs, especially when it heterodimerizes with RXR, we aimed to test the potential antidiabetic effect of phytol (250 mg/kg), the natural precursor of phytanic acid, a RXR ligand and/or pioglitazone (5 mg/kg) to diabetic insulin-resistant rats. Regarding the molecular docking simulation on PPARγ, phytanic acid, rather than phytol, showed a binding mode that mimics the crystal orientation of rosiglitazone and pioglitazone, forming H bonds with the same amino acids (S289, H 323, H 449 and Y 473), and the least energy level, which emphasizes their importance for PPARγ molecular recognition, activation, hence antidiabetic activity. In addition, docking on the RXRα/PPARγ heterodimer, revealed that phytanic acid has higher binding affinity and lesser energy score on RXRα, compared to the original ligand, retinoic acid. Phytanic acid binds by 3H bonds and shares retinoic acid in arginine (R 316). These results were further supported biochemically, where oral phytol and/or pioglitazone (5 mg/kg) improved significantly glucose homeostasis, lipid panel, raised serum adiponectin level and lowered TNF-α, reaching in most cases the effect of the 10 mg/kg pioglitazone. The study concluded that the insulin sensitizing/anti-diabetic effect of phytol is mediated by partly from activation of nuclear receptors and heterodimerization of RXR with PPARγ by phytanic acid.

## Introduction

Type-2 diabetes and obesity are rampant metabolic disorders in modern society [1], originating from the slothful lifestyle along with the high consumption of westernized diet which is rich in dietary fat content and fructose in soft drinks [2]. Furthermore, excessive accumulation of triglycerides (TGs) and certain fatty acid derivatives in skeletal muscles and other tissues mediate many of the adverse effects of insulin resistance syndrome [3], which is associated with a constellation of pathological metabolic disorders [1].

Current antidiabetics, including insulin secretagogues, insulin sensitizers or their combination, provide a sensible therapeutic approach [4]. Thiazolidinediones (TZDs), the insulin sensitizers, act by activating the nuclear receptors peroxisome proliferator–activated receptors (PPARs) [5] and selectively PPARγ subtype. The latter heterodimerizes with retinoid X receptor (RXR), to trigger transcription factors in adipocytes and skeletal muscles [6]. Albeit the positive effect of oral hypoglycemics, several side effects can develop, and over time, some patients lose response towards them; factors that could hinder their efficacy and safety. Accordingly, attention has been driven towards finding RXR agonists that can safely and effectively potentiate or replace TZDs. Synthetic ligands for RXR-dubbed as ‘rexinoids’- function as TZDs where they ameliorate insulin resistance [7], lower hyperglycemia in type-2 diabetes and obesity [8] and enhance pre-adipocytes differentiation [9]. Moreover, the combined effect of rexinoids with TZDs was greater than either agent alone [7].

Phytol, a branched-chain fatty alcohol present as part of the chlorophyll pigment, is released only in the ruminants' digestive system, presumably by bacteria present in their gut [10], hence, it is present in the animals' adipose tissues and dairy products at a relatively high amounts [11]. Gloerich et al. [12] reported increased levels of phytol and its metabolites, viz., phytenic, phytanic and prystanic acids, in plasma and liver of mice fed phytol enriched diet, and suggested that once phytol is taken up by the body, it is transported to the liver to be metabolized into phytanic acid through three enzymatic steps [13], which are believed to be under the control of PPARα [12]. In humans and mammals phytanic acid appears as an oxidized product of phytol following ingestion of fat-containing foods of animal origin or vegetables and their absorption in small intestine. Phytol, therefore, is a precursor of the natural rexinoid phytanic acid that, besides triggering RXR, it activates the full spectrum of PPARs [14]. It also boosts the transcriptional activity of the PPAR/RXR heterodimers [3] and other nuclear receptors, viz., retinoic acid receptor (RAR) [15] where phytol and its metabolite, potentiate the teratogenic effects induced by a synthetic RARα agonist in mice.

Previous *in vitro* studies reported that phytanic acid activates several PPARs subtypes [14]–[16], and PPARγ agonists are widely used in the treatment of type 2 diabetes. Therefore, the present work pointed toward verifying the potential antidiabetic effect of phytol by comparing it, and its metabolite, with the TZDs using molecular docking techniques (Molsoft ICM 3.4-8C program) in an unprecedented attempt to visualize the orientation, binding modes and affinities of these compounds to the active sites of their receptor(s). The goal extended also to validate the *in vivo* effect of phytol alone and its modulatory action on pioglitazone using an insulin-resistant diabetic rat model [17].

## Materials and Methods

### 1. Method of the drug modeling studies

All docking studies were performed using “Internal Coordinate Mechanics (Molsoft ICM 3.4-8C)”. Molsoft, a flexible docking program, enables the prediction of favorable protein-ligand complex structures with reasonable accuracy and speed [18]. ICM docking is currently the most accurate predictive tool of the binding geometry of biologically active compounds [18], [19], [20].

Phytol, phytanic acid, and pioglitazone were first docked on the crystal structures of PPARγ (2PRG), which is originally complexed with its ligand rosiglitazone, as found in RCSB Protein Data Bank (PDB entry 2PRG) [21]. In addition, we performed another docking study of phytanic acid and/or pioglitazone into the crystal structures of the RXRα/PPARγ (1FM6) heterodimer, that was available through the RCSB Protein Data Bank (PDB entry 1FM6) [22], and contains two binding sites for two ligands. One active site is in complex with the 9-cis retinoic acid (9cRAR) which is a selective agonist to RXRα, and the other is complexed with rosigitazone which is the binding ligand of PPARγ receptor.

#### 1.A. Preparation of small molecules

ChemDraw 3D structures were constructed using Chem 3D ultra 8.0 software [Molecular Modeling and Analysis; Cambridge Soft Corporation, USA (2004)], and then they were energetically minimized using MOPAC (semi-empirical quantum mechanics), Jop Type with 100 iterations and minimum RMS gradient of 0.01, and saved as MDL MolFile (*.mol).

#### 1.B. Generation of ligand and protein structures

The crystal structures of target protein PPARγ (2PRG) and RXRα/PPARγ heterodimer (1FM6) active sites were retrieved from the Protein Data Bank (http://www.rcsb.org/pdb/welcome.do). All bound waters, ligands and cofactors were removed from the protein. The amino acids of the binding site where defined using data in pdbsum (http://www.ebi.ac.uk/thoronton-srv/databases/pdbsum).

#### 1.C. Docking using Molsoft ICM 3.4-8C program

The docking procedure was accomplished in the following sequence:

- Conversion of PDB file into an ICM object: This conversion involves addition of hydrogen bonds, assignment of atoms' types, and charges from the residue templates.
- Docking of ICM small molecule in the following sequence:

*Setup Docking Project*: Set project name, setup the receptor, review and adjust binding site, and make **receptor maps**

*Start docking simulation*

*Display of the result*

ICM stochastic global optimization algorithm attempts to find the global minimum of the energy function that includes five grid potentials describing interaction of the flexible ligand with the receptor and internal conformational energy of the ligand, during this process a stack of alternative low energy conformations is saved. The mode of interaction of rosiglitazone within 2PRG, and 1FM6 was used as a standard docked model. All agonists were compared according to the best binding free energy (minimum) obtained among all the run.

### 2. Materials and method of the experimental model

#### 2.A. Drugs and chemicals

Streptozotocin (STZ) and phytol were purchased from Sigma Aldrich Co. (St Louis, MO, USA), pioglitazone from Medical Union Pharmaceutical Co. (Ismailia, Egypt), and long acting insulin (Monotard) from Eli Lilly (USA). Cholesterol and lard were obtained from commercial sources, and fructose from El-Nasr Chemical-Co. (AbouZaabal, Cairo, Egypt). Phytol was diluted with cottonseed oil and pioglitazone was suspended in 1% Na-CMC.

#### 2.B. Animals

Adult male Wistar albino rats (80–120 g; National Research Center Laboratory, Cairo, Egypt) were oused (three rats/cage), in polypropylene cages, kept on a 12 hr light/dark cycle, and constant environmental conditions. Animals were fed commercially available normal pellet and water *ad libitum*, prior to the dietary manipulation. Experimental design and animal handling were performed according to the guidelines of the Animal Care and Use and after approval of the Ethics Committee of Faculty of Pharmacy, Cairo, Egypt.

#### 2.C. Development of diabetic insulin-resistant rats

Rats were divided into two dietary regimen-groups, normal fat diet (NFD; n = 20), and high fat diet (HFD; n = 80) with fructose (20%) in drinking water, until the rats' body weights reached 220±40 g. The composition of NPD [3.15 kcal/g; fat (5%), protein as casein (21%), carbohydrate as starch (60%), fibers (3%), vitamins and minerals (4.5%)] and this group served as normal control. While the HFD was[4.1 kcal/g; fructose (60%), lard (10%), Casein (20.7%), Cellulose (4.2%), Mineral Mix (3.5%), Vitamin Mix (1%), Calcium Carbonate (0.3%) and DL-Methionine (0.3%)]. During the 6^th^ week, animals received a daily single dose of Monotard (0.5 IU/kg, i.p). By the end of this week, and after an overnight food deprivation, HFD fed rats received freshly prepared STZ in citrate buffer (35 mg/kg, i.p, single dose) [17]. NFD group received either vehicle or same dose of STZ and the results recorded in table 1. As no significant difference was reported between both groups, the NFD receiving vehicle was considered as the normal reference group.

**Table 1:** Effect of STZ (35 mg/kg, i.p.) on NPD -fed rats.

Parameters	NPD	NPD+STZ
Glucose(mg/dl)	93±10	110±15
Insulin(µIU/ml)	11±2	9.5±1
TG(mg/dl)	50+6	57±7
TC(mg/dl)	100±12	80±9.5
HDL-C(mg/dl)	40±3.5	38±4
LDL-C(mg/dl)	47±5.5	50±5

Values are means±SD; NPD: Normal pellet diet.

Periodic estimation of body weight (BW) and levels of fasting serum glucose, triglycerides (TGs), total cholesterol (TC) and insulin were determined. Only animals with persistent blood glucose levels between 200–350 mg/dl, hyperinsulinemia, and hypercholesterolemia, for 7 days after STZ, were considered diabetic insulin resistant and were used in the study and permitted HFD and fructose during the treatment period.

#### 2.D. Intra-peritoneal glucose (GTT) and insulin glucose (IGTT) tolerance tests

One week after STZ administration, two groups (n = 6) of 6 hr-food deprived diabetic rats, were given glucose (2 g/kg, ip) [17] without (GTT) and with (IGTT) insulin injection (0.4 I.U/kg; i.p), with a slight modification of Levy et al. [23] to suit animals' sensitivity. Droplets of blood from the tail vein were withdrawn (under brief ether anesthesia) every 30 min., and up to 120 min. to evaluate the resulting glucose concentrations. Two groups of non-diabetic rats (n = 6) were similarly tested.

#### 2.E. Effect of phytol and pioglitazone on diabetic insulin-resistant rats

One week after STZ administration, five groups of diabetic rats (n = 7) were given daily oral doses of cottonseed oil (0.5 ml/kg, group DV), phytol (250 mg/kg, group DP_h_), pioglitazone (5 and 10 mg/kg, groups DP_5_ and DP_10_) or a combination of phytol with pioglitazone (5 mg/kg) (group DP_h_P_5_). All drug regimens continued for 2 weeks, and the last dose of any treatment was given 24 hours before rats were euthanized. Animals were fasted 18 hours before the time of carnage, to minimize feeding induced variations in lipid and glucose pattern.

#### 2.F. Collection of samples for analysis

Before killing, rats were weighed, then euthanized and serum was separated from the collected blood to assess levels of glucose, fructosamine, insulin, TGs, TC, LDL-C, HDL-C, TNF-α and adiponectin, as well as, the activity of ALT. Rats were then euthanized and liver, visceral fat (VF) and epididymal fat (EF) were carefully dissected out, weighed and their weights were expressed as a ratio of body weight (BW) multiplied by a factor of 100. Glucose and lipid profiles were assessed colorimetrically using commercially available kits, while HOMA-index was calculated according to Matthews et al. [24]. RIA techniques were adopted to determine concentrations of insulin and adiponectin, whereas TNF-α was measured by an ELISA kit (R&D Systems, Minneapolis, USA). Liver specimens were used to determine TGs and TC after lipid extraction [25].

#### 2.G. Measurement of Phytanic Acid

Extraction and derivitization of serum samples for analysis of phytanic acid was performed using gas liquid chromatography/mass spectroscopy analysis. Serum was subjected to saponification using ethanolic KOH with triheneicosanoic acid as internal standard. The free fatty acid moieties were extracted into hexane and converted to the more volatile fatty acid methyl esters (FAME), which were extracted and injected onto a GLC column. The separation of FAME and the analysis of phytanic acid was performed on a Trace 2000 GC from ThermoQuest with an AS 2000 autosampler and a Finnigan trace mass spec. Phytanic acid methyl ester as a reference standard was purchased from Sigma-Aldrich [26].

The analysis results reveal that the administration of 250 mg phytol to the experimental rats released 18 µg/ml±2.5 phytanic acid in the serum.

#### 2.H. Statistical analysis

Results are expressed as means ± SD and differences between groups were tested for significance using analysis of variance (ANOVA), followed by Tukey post hoc test, *P*<0.05. To test for an interaction between individual treatments when given in combination, a factorial design test is used. Correlation coefficient (r) between serum glucose, insulin and HOMA-index with serum TNF-α, adiponectin, ALT and VFW/BW ratio was carried out in untreated and treated hyperglycemic animals using linear regression analysis.

## Results

**Figure 1:**
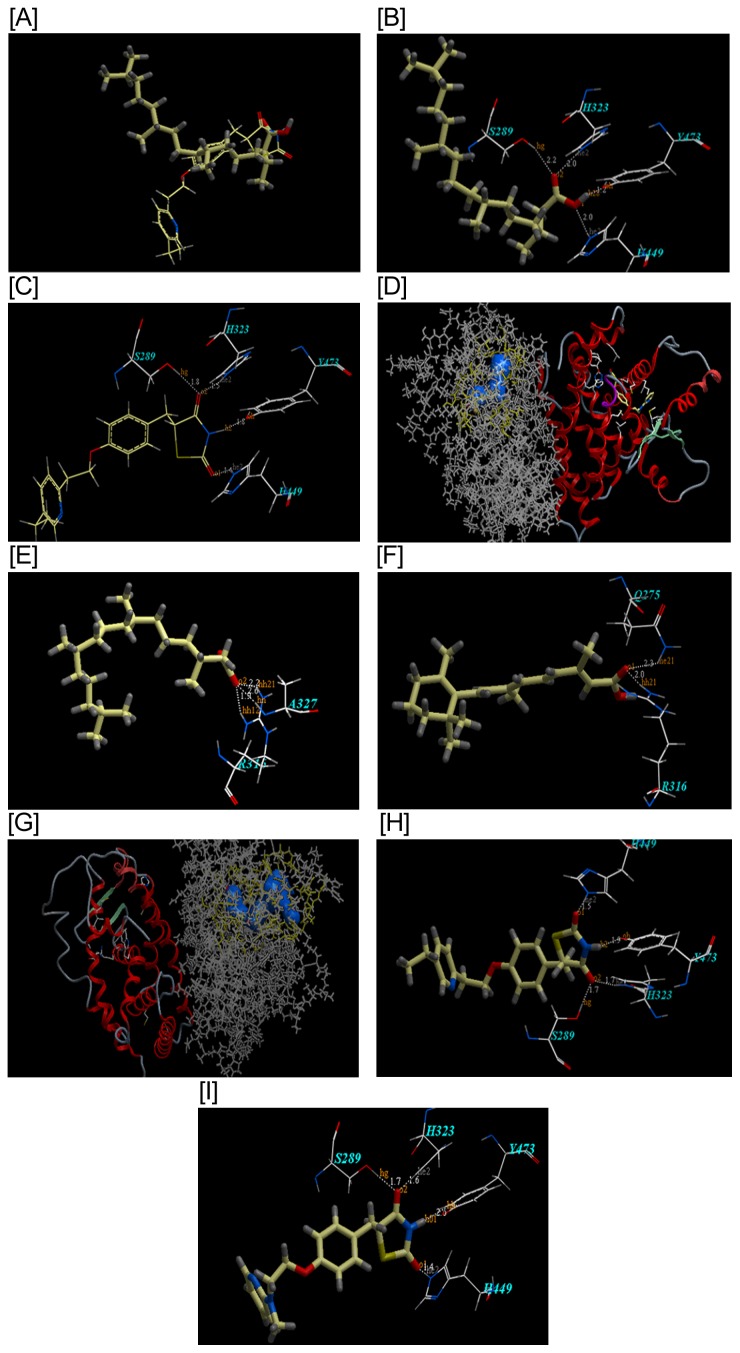
The docking modes of phytanic acid in the crystal structures of PPARγ binding protein and RXR receptor in terms of orientation, fitting and binding affinity as compared with phytol rosiglitazone, pioglitazone and retinoic acid. **A**: The superimposition of phytanic acid (wire and stick) and pioglitazone (wire) into the binding site of PPAR γ, (for interpretation of the references to color in the text, the reader is referred to the web version of this article). **B**: The binding mode of phytanic acid into the binding site of PPAR γ. It exhibits an ICM score of (−111.09) and forms 4 hydrogen bonds shown as white dotted lines (Table 2). These encompass one hydrogen bond between O of carbonyl moiety with S-289 of distance 2.17 A; another bond between O of CO with H323 of distance 2.03 and another two H bonds between O of OH with H- 449 of distance 1.89 A and between H of OH with Y 473 of distances 1.22A. (For interpretation of the references to color in the text, the reader is referred to the web version of this article). **C**: The binding mode of pioglitazone into the binding site of PPAR γ. It exhibits an ICM score of (−105.43) and forms 4 hydrogen bonds shown as white dotted lines (Table 2). These encompass two hydrogen bonds between O of CO with S 289 and H323 of distance 1.20 A and 0.70 A, respectively and another two between H of NH with Y-473 and O of CO with H-449 of distance 1.80 A and 2.76 A, respectively. (For interpretation of the references to color in the text, the reader is referred to the web version of this article). **D**: The binding mode of phytanic acid docked in RXR α binding site (left), while PPAR γ is bound with its ligand rosiglitazone (right). **E**: The binding mode of phytanic acid into the binding site of RXRα. It exhibits an ICM score of −114.75 and forms 3 hydrogen bonds shown as white dotted lines (Table 3). These include two hydrogen bonds between O of 2-oxo of carboxylic acid moiety with R-316 of distance 1.92 A and 2.15 A, and another bond with A-327of distance 2.63 A. (For interpretation of the references to color in the text, the reader is referred to the web version of this article). **F**: The binding mode of retinoic acid into the binding site of RXR α revealed an ICM score of (−86.79) and the existence of 2 hydrogen bonds shown as white dotted lines (Table 3) between O of 2-oxo moiety with R-316, and Q-275 of distance 1.96 A and 2.32 A, respectively. (For interpretation of the references to color in the text, the reader is referred to the web version of this article). **G**: The binding mode of pioglitazone docked in PPARγ binding site, while RXR α with its ligand 9-cis retinoic acid. **H**: The binding mode of pioglitazone into the binding site of PPARγ revealed having an ICM score of −106.37 and the existence of 4 hydrogen bonds shown as white dotted lines (Table 3). Two hydrogen bonds are shown between O of 2-oxo moiety with S-289, and H-323 of distance 1.66 A and 1.66 A, respectively. One H bond between another O of 4-oxo moiety with H-449 of distance 1.47 A and another H bond between H of NH of TZD with Y-473 of distances 1.89 A. (For interpretation of the references to color in the text, the reader is referred to the web version of this article). **I**: The binding mode of rosiglitazone into the binding site of PPAR γ revealed having an ICM score of −104.37 and the existence of 4 hydrogen bonds shown as white dotted lines (Table 3). Two hydrogen bonds exist between O of 2-oxo moiety with S-289, and H-323 of distance 1.66 A and 1.64 A, respectively. One H bond exists between another O of 4-oxo moiety with H-449 of distance 1.43 A and another H bond between H of NH of TZD with Y-473 of distances 1.79 A. (For interpretation of the references to color in the text, the reader is referred to the web version of this article).

Table 2, Figure 1A–C depict the docking results of phytanic acid in the crystal structures of PPARγ binding protein (2PRG) in terms of orientation, fitting and binding affinity as compared with rosiglitazone, pioglitazone and phytol. Docking simulations showed a binding mode of phytanic acid very similar to the crystal orientation of the TZD head group of rosiglitazone, and pioglitazone as well. Phytanic acid fitted with the PPARγ ligand binding domain interacting via hydrogen bonding with Ser- 289, His-323, His-449, and Tyr-473. Interestingly, the ICM score values of phytanic acid on PPARγ proved that it has the least energy levels with the highest binding affinity compared to those of pioglitazone and rosiglitazone (ICM score: −111.09, −105.43 and −103.26, respectively). On the other hand, phytol complexed only to two amino acids, viz., Ser-289 and His-323 by two hydrogen bonds, and it showed the highest energy level with the least binding affinity (ICM score: −102.57). Table 3 and figures 1D–F and G–I show the second docking results, which revealed that phytanic acid on RXRα, has a higher binding affinity and lesser energy score as compared to retinoic acid, the natural ligand (ICM scores: −114.75 for phytanic acid vs −86.79 for retinoic acid). While phytanic acid was complexed to RXR receptor via 3 H bonds (2R and 1A), it shared retinoic acid in arginine R 316. The pioglitazone binding affinities and complexing properties of the heterodimer mimicked those of rosiglitazone and the binding to the PPARγ single receptor, having an ICM score of −106.37 vs −104.37for rosiglitazone. Pioglitazone again bound to the receptor by complexing to the same four amino acids (S, H, H and Y) via 4 H bonds.

**Table 2:** ICM scores of phytol, phytanic acid, pioglitazone, and rosiglitazone docked in PPAR**γ** of the 2 PRG crystal structure binding sites and hydrogen bonds formed with amino acid residues.

Compounds	ICM scores	No. of hydrogen bonds	Involved group of amino acid	Atom of ligand involved	Length of hydrogen bond
Phytol	−102.57	2	S289 ….. hg	O of OH	1.85 A
			H323 …..he2	O of OH	1.86 A
Phytanic acid	−111.09	4	S289 …. hg	O of CO	2.17 A
			H323 …..he2	O of CO	2.03 A
			H449 …. he2	O of OH	1.89 A
			Y473 … oh	H of OH	1.22 A
Pioglitazone	−105.43	4	S289 …. hg	O of CO (TZD)	1.20 A
			H323…. he2	O of CO (TZD)	0.70 A
			H449 ….. he2	O of CO (TZD)	2.76 A
			Y473…. oh	H of NH (TZD)	1.80 A
Rosiglitazone	−103.26	4	S289….. hg	O of CO (TZD)	1.79 A
			H323….. he2	O of CO (TZD)	1.59A
			H449…. he2	O of OH (TZD)	1.39 A
			Y473….. oh	H of NH (TZD)	1.81 A

**Table 3:** ICM scores of phytanic acid, retinoic acid, pioglitazone, and rosiglitazone docked in 1FM6 crystal structure binding site and hydrogen bonds formed with amino acid residues.

Compounds	ICM scores	No. of hydrogen bonds	Involved group of amino acid	Atom of ligand involved	Length of hydrogen bond
Phytanic acid	−114.75	3	R316 … hh12	O of CO	1.92 A
			R316….. hh21	O of CO	2.15 A
			A327……hn	O of CO	2.63 A
Retinoic acid	−86.79	2	R316..hh21	O of CO	1.96 A
			Q275…..hh21	O of CO	2.32 A
Pioglitazone	−106.37	4	S289….hg	O of CO (TZD)	1.66 A
			H323…he2	O of CO (TZD)	1.66 A
			H449….he2	O of CO (TZD)	1.47 A
			Y473…oh	H of NH (TZD)	1.89 A
Rosiglitazone	−104.37	4	S289….hg	O of CO (TZD)	1.66 A
			H323…he2	O of CO (TZD)	1.64 A
			H449….he2	O of CO (TZD)	1.43 A
			Y473…oh	H of NH (TZD)	1.79 A

**Figure 2:**
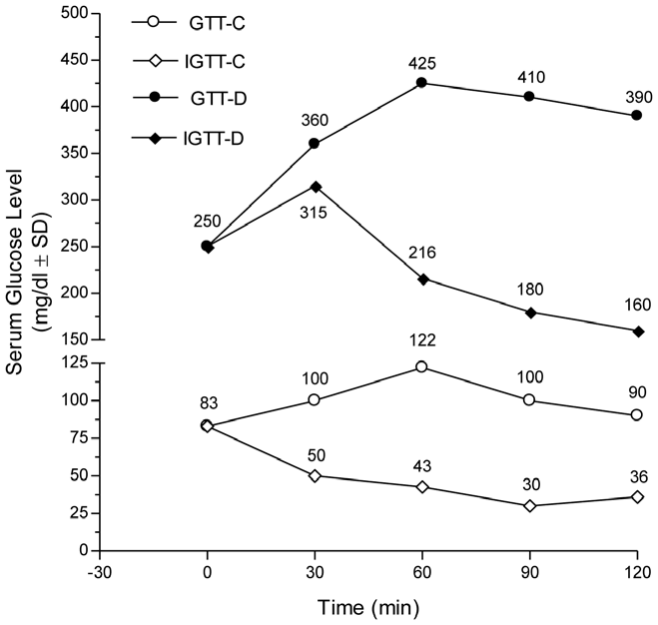
The glucose tolerance curve of the experimental groups. The glucose tolerance curve depicts the effect of 2 g/kg, ip, glucose without (GTT, ○), and with concurrent insulin injection (0.4 IU/kg, ip) (IGTT, ◊) in serum of normal control (hollow-C) and insulin-resistant rats (solid-D), after 0, 30, 60, 90, and 120 min. Values are means (± S.D.) of six animals. As compared with the normal control group, P≤0.05 (unpaired Student's t test).

**Figure 3:**
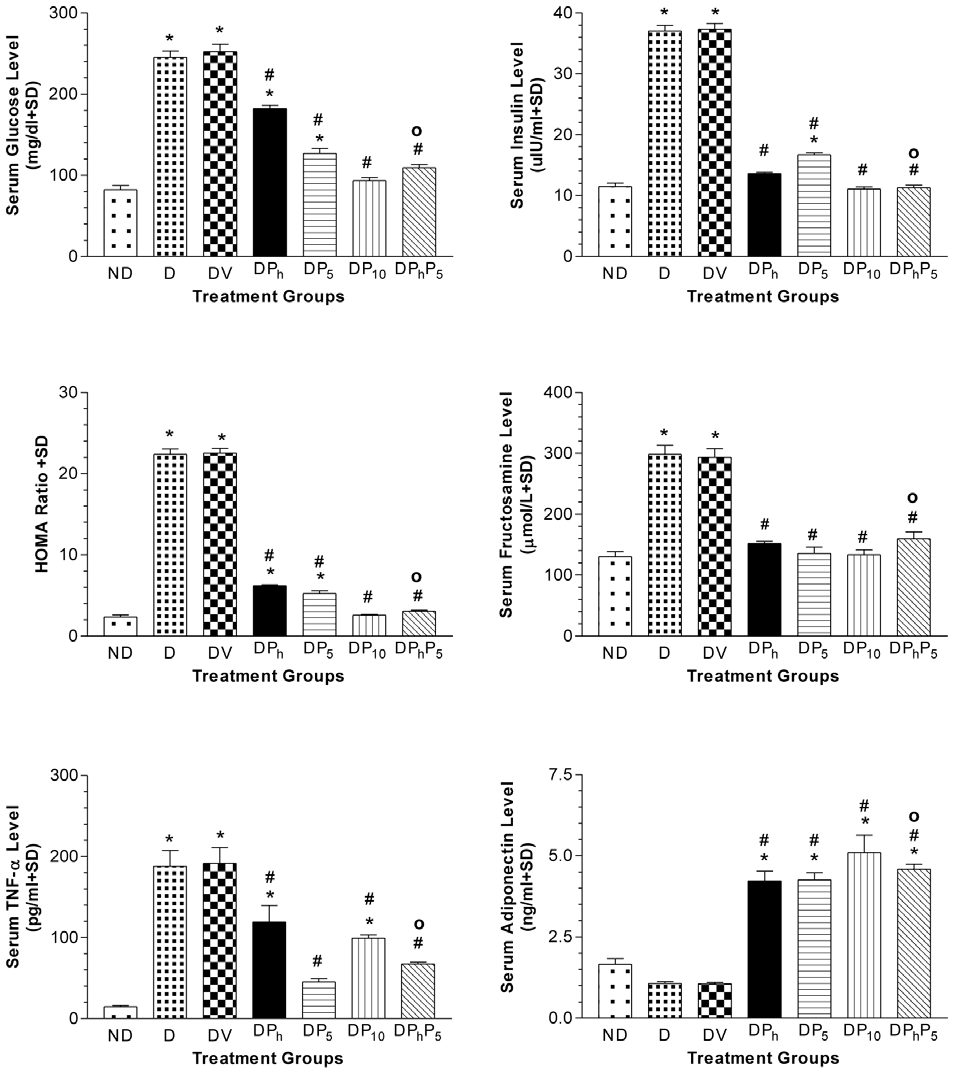
Effect of diabetes (DV) and different oral drug regimens on glucose homeostasis indicators. Effect of diabetes (DV) and different oral drug regimens, viz., phytol (DP_h_, 250 mg/kg); pioglitazone (DP_5_, 5 mg/kg); pioglitazone (DP_10_, 10 mg/kg); phytol and pioglitazone (DP_h_P_5_); on serum levels of glucose, insulin, fructosamine, TNF-α, adiponectin and insulin resistance (HOMA-ratio) (mean of 7 animals ± S.D). As compared with non-diabetic [ND] (*) and diabetic [DV] (#) groups using one way ANOVA followed by Tukey post hoc test, *P*<0.05. (^Ο^) Significant interaction when P_h_ and P_5_ were combined using Factorial Design.

**Figure 4:**
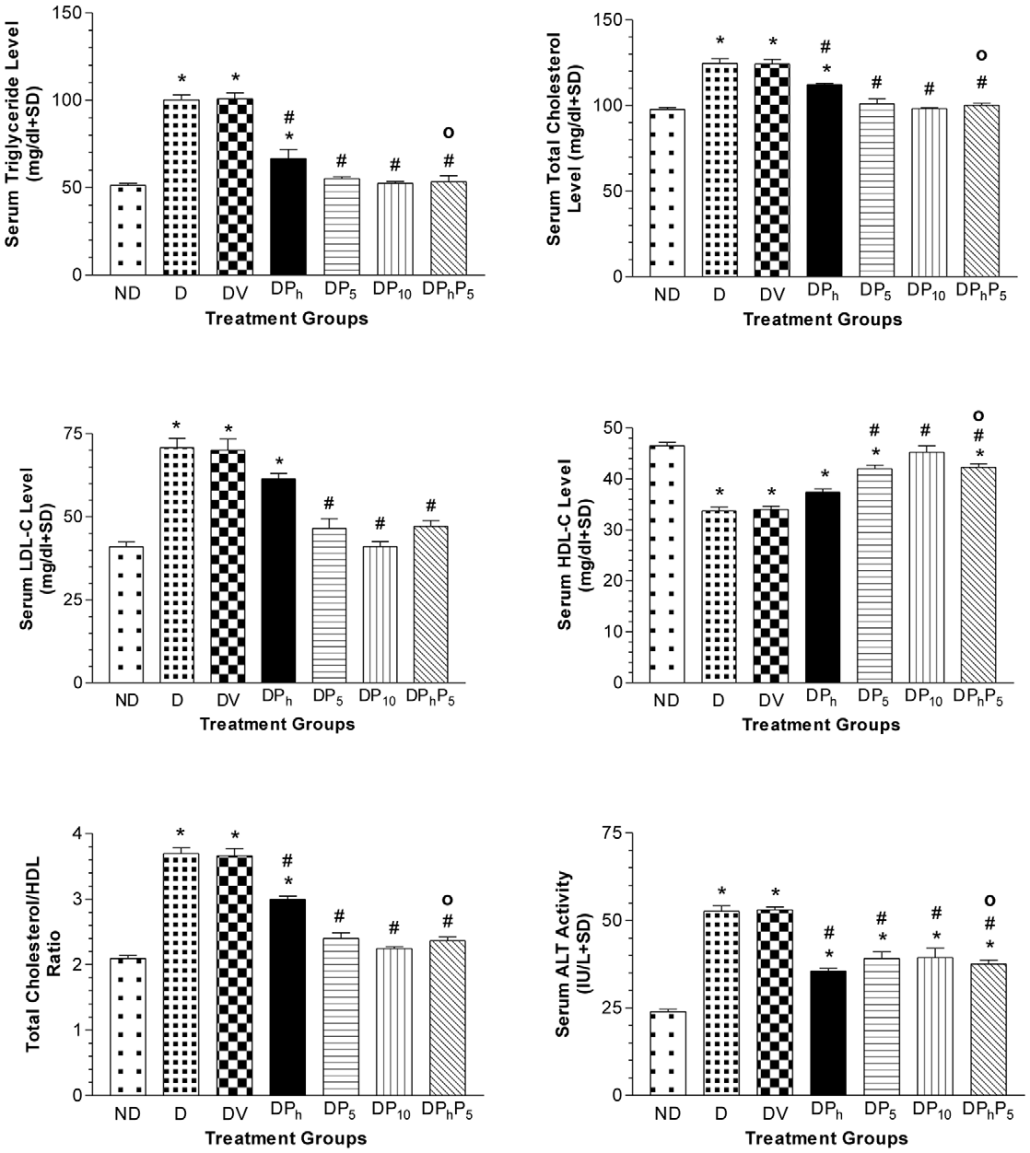
Effect of diabetes (DV) and different oral drug regimens on the serum lipid profile. Effect of diabetes (DV) and different oral drug regimens, viz., phytol (DP_h_, 250 mg/kg); pioglitazone (DP_5_, 5 mg/kg); pioglitazone (DP_10_, 10 mg/kg); phytol and pioglitazone (DP_h_P_5_); on serum levels of triglycerides, total cholesterol, LDL-C, HDL-C, ALT and TC/HDL ratio (mean of 7 animals ± S.D). As compared with non-diabetic [ND] (*) and diabetic [DV] (#) groups using one way ANOVA followed by Tukey post hoc test, *P*<0.05. (^Ο^) Significant interaction when P_h_ and P_5_ were combined using Factorial Design.

**Figure 5:**
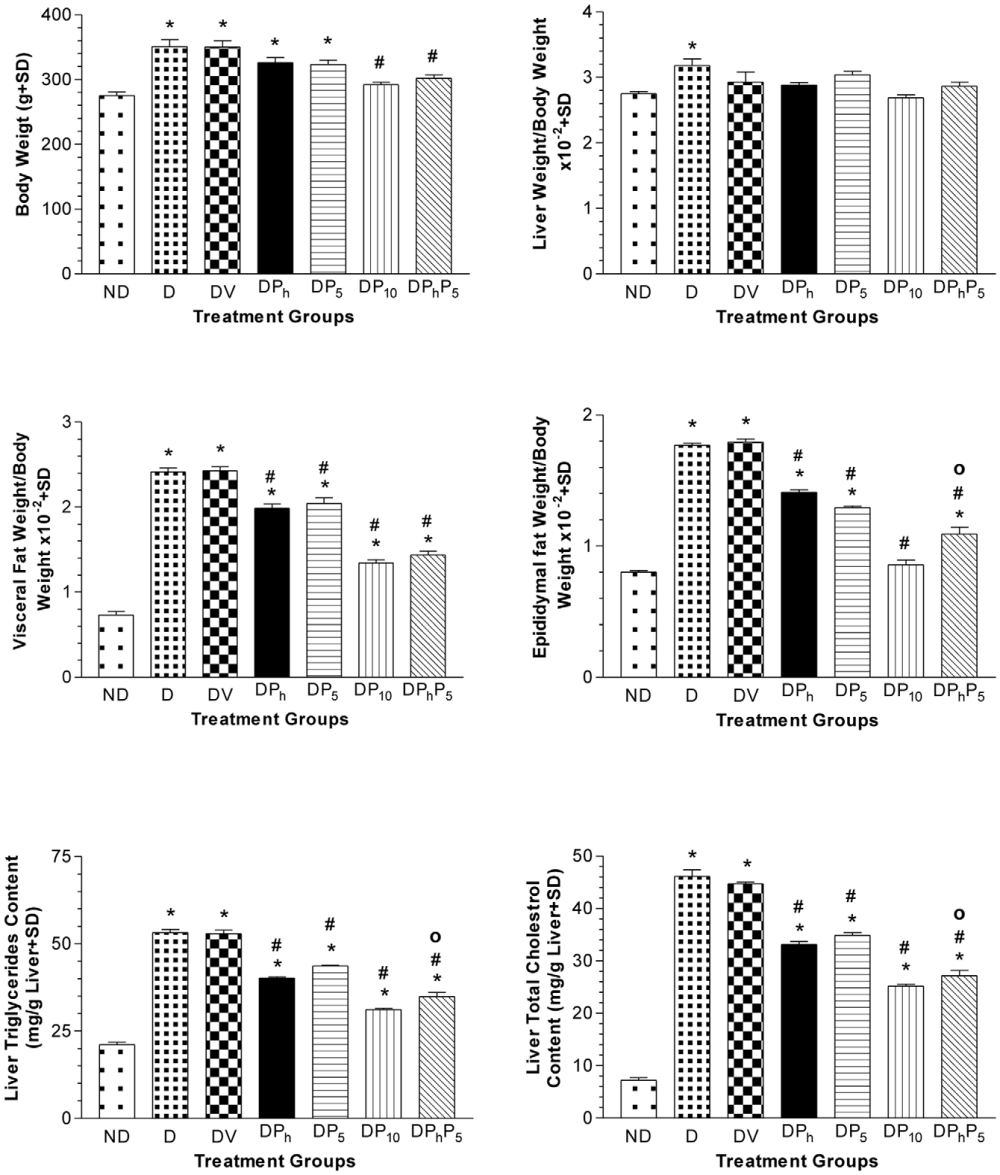
Effect of diabetes (DV) and different oral drug regimens on liver weight:, visceral fat:, and epidydimal fat:body weight ratios and on hepatic triglycerides and total cholesterol contents. Effect of diabetes (DV) and different oral drug regimens, viz., phytol (DP_h_, 250 mg/kg); pioglitazone (DP_5_, 5 mg/kg); pioglitazone (DP_10_, 10 mg/kg); phytol and pioglitazone (DP_h_P_5_); on body weight, ratios of liver weight/body weight, visceral fat weight/body weight, and epidydimal weight/body weight, and contents of liver triglycerides and total cholesterol(mean of 7 animals ± S.D). As compared with non-diabetic [ND] (*) and diabetic [DV] (#) groups using one way ANOVA followed by Tukey post hoc test, *P*<0.05. (^Ο^) Significant interaction when P_h_ and P_5_ were combined using Factorial Design.

Regarding the biochemical results, comparisons were related to the DV group since no significant difference between DV and D groups was reported. GTT and IGTT in diabetic and non-diabetic rats were shown in figure 2. Glucose injection caused a marked steady rise of serum glucose level in diabetic rats over that of non-diabetic ones along the tested period. Nevertheless, the co-administration of insulin with glucose increased serum glucose level only at the 30 minutes point followed by a steady decrease in diabetic rats. However, in non-diabetic rats, the same pattern was observed, but without initial rise at the 30 minutes point. Compared to normal group, the levels of glucose, fructosamine, and insulin, along with HOMA index and TNF-α were elevated by 3.0, 2.3, 3.2, 9.5 and 13 folds, respectively in diabetic rats; however, adiponectin level was significantly lower than the control group only when using Student's t test (data not shown) (Fig. 3). Serum TG, TC, LDL-C and ALT were elevated markedly in diabetic animals (Fig. 4), while HDL-C was 30% lower as compared to non-diabetic group, with a consequent increase in TC/HDL ratio (76%). Diabetic rats gained more BW (27%), LW (30%) and LW/BW ratio (15%) (Fig. 5). In addition, VFW/BW and EFW/BW ratios were elevated by 3.3 and 2.2 folds, respectively, as well as the liver content of TGs (2.5 folds) and TC (6.4 folds).

Phytol repressed insulin and fructosamine levels back to normal, decreased those of glucose, HOMA-index and TNF-α, as compared to control group and increased adiponectin level by 4 folds (Fig. 3). Phytol also reduced serum levels of TGs (34%), TC (10%), ALT (33%), and TC/HDL-C ratio (18%) as presented in figure 4. It also leveled off LW (22%), VFW/BW (18%) and EFW/BW ratios (21%), along with liver contents of TGs (24%) and TC (26%), as compared to diabetic non-treated rats (Fig. 5). Groups DP_5_ and DP_10_ showed a reduced glucose (50%; 63%), insulin (55%; 70%), fructosamine (54%; 55%) and TNF-α (76%; 48%) levels, as well as HOMA-index (77%; 89%), whilst adiponectin was raised by 4 and 4.8 folds, respectively (Fig. 3). Pioglitazone (5 mg/kg) improved serum lipid homeostasis and ALT activity, effects that reached to normal levels in DP_10_group (Fig. 4). Both doses of pioglitazone reduced hepatic TGs (17%; 41%) and TC (22%; 44%), as well as, LW (17%; 23%), VFW/BW ratio (16%; 45%), and EFW/BW ratio (28%; 52%), while BW was lowered only in DP_10_ group (Fig. 5).

DP_h_P_5_ group showed recovered glucose and lipid homeostasis, insulin, HOMA-index, TNF-α and ALT to reach values virtually close to those of the control non-diabetic rats (Fig. 3 and 4). In addition, this combination raised serum levels of adiponectin (4.3 folds) (Fig. 3) and HDL-C (24%), (Fig. 4), while reduced VFW/BW and EFW/BW ratios, along with BW, LW and liver contents of TGs and TC (Fig. 5).

To detect the possible interactions between phytol and pioglitazone, the results of DV, DP_h_, DP_5_ and DP_h_P_5_ groups were statistically analyzed using factorial design. There was an additive effect of the combination compared with individual treatments only on BW and VFW/BW ratio. Although in all other parameters measured, there was a significant interaction, where the combined effect was higher than that of either individual treatment, yet this interaction did not reach an additive effect statistically. Using linear regression analysis, glucose, insulin and HOMA-index correlate positively with TNF-α, ALT and VFW/BW ratio and negatively with adiponectin, *P*<0.001(Table 4).

**Table 4:** Correlation coefficient (*r*) between serum glucose, insulin and HOMA-R with TNF-α, adiponectin, ALT and visceral fat weight/body weight ratio.

	TNF-α	Adiponectin	ALT	Visceral fat/BWT
Glucose	O.7089	−0.8567	0.6915	0.8664
	P<0.001	P<0.001	P<0.001	P<0.001
Insulin	0.7299	−0.9163	0.8255	0.8345
	P<0.001	P<0.001	P<0.001	P<0.001
HOMA-R	0.7590	−0.9196	0.8264	0.8319
	P<0.001	P<0.001	P<0.001	P<0.001

Correlation was carried out in untreated and treated hyperglycemic animals only.

## Discussion

To the best of the author's knowledge, the present work is the first in vivo study to verify the antidiabetic/insulin sensitizing action of phytol. The docking study proved that phytanic acid has high affinity to interact with PPARγ in a pattern similar to that of the TZD agonists, via 4 hydrogen bonding with the same amino acids, viz., Ser- 289, His-323, His-449, and, Tyr-473. Hence, these 4 amino acids are considered indispensable for the PPARγ molecular recognition, activation and antidiabetic biological activity. Moreover, the current finding emphasized that phytanic acid complexes to RXR with lesser energy than retinoic acid, the natural ligand, and shared retinoic acid in arginine R 316 which highlights the vital role of arginine R 316 for the dimer activity.

Therefore, the phytol-induced glucose homeostasis salvage is partly attributed to the ability of phytanic acid to boost the expression of transcriptional activity of PPARs/RXR heterodimers that regulates several genes' expression [3], via the binding to PPAR-responsive element (PPRE). This insinuates that either partner, RXR or PPARγ, can regulate the transcriptional activity by interacting with its own ligand. In this essence, phytol mimics its synthetic candidates, which through activating RXR could have a marked impact on whole body metabolism, including insulin-sensitization and improved glycemic control, in a comparable action to TZDs.

Phytanic acid by activating RXR and/or PPARγ can activate GLUT2 gene, and glucokinase mRNA, effects that facilitate hepatic glucose influx [14], [27]. In addition, agonists of this nuclear receptor increase the expression and translocation of GLUT4 in adipocytes, and the catabolism of glucose along with the decrease in hepatic glucose output [28].

Furthermore, the dyslipidemic effect of phytol, which coincides relatively with a previous study [29] may be attributed again to the phytanic acid-induced activation of PPARs/RXR heterodimerization, a dimer that induces various genes involved in lipid homeostasis [30]. In the presence of decreased glucose level [28], stimulation of PPARα, enhances lipid metabolism through up-regulating enzymes involved in β-oxidative degradation of fatty acids [14], [28], a critical fate in regulation of VLDL synthesis [31]. PPARα-dependent and -independent β-oxidation enzymes are induced by phytol feeding, as reported previously [12]. The ability of phytol to lower FFAs [12] could improve glucose profile by enhancing hepatic glucose uptake, decreasing gluconeogenesis, suppressing hepatic glucose production, and mobilizing lipids from muscle, leading to enhanced muscle sensitivity to insulin, and suppressing delivery of TG's substrates to the liver [31].

Besides its action on redressing altered glucose panel and improving dyslipidemic state observed in this model, phytol also elevated adiponectin and decreased that of TNF-α and ALT; results that play a role in its antidiabetic effect. Moreover, visceral and epidydimal fat contents correlate positively with insulin resistance [32], hence, phytol-mediated decrease in VFW/and EFW/BW ratios, offers another mechanism for improved insulin sensitivity.

White adipose tissue (WAT), represented herein by epidydimal fat content, participates in the induction of whole-body insulin resistance, partly by the macrophage infiltration-induced chronic inflammation of WAT and/or the release of TNF-α, which is highly expressed in WAT [33]. TNF-α is a critical mediator in insulin resistance induction [34], [35], inhibition of which by phytol could convey the improvement of insulin sensitivity along with glucose disposal.

In the current study, phytol boosted the adiponectin level, another adipocytokine that is closely associated with improved insulin sensitivity [36]. The adiponectin promoter contains a functional PPRE to which PPARγ/RXR heterodimer binds directly and increases its activity in adipocytes [37]. Our docking results confirmed the binding of phytanic acid with the RXR in the heterodimer; a finding that can verify the phytol-induced increase in adiponectin. Adiponectin antidiabetic properties result from enhancement of insulin-induced tyrosine phosphorylation of the IR [36], activation of IRS-1-mediated phosphatidylinositol-3 kinase (PI-3K) and glucose uptake in hepatic and skeletal muscle cells [37]. Besides, it enhances muscle β-oxidation, stimulates glucose utilization, suppresses hepatic glucose production [38], and decreases TNF-α level [39].

Another possible mechanism for the insulin sensitizing/anti-diabetic effect of phytol is its ability to lower ALT activity; an enzyme found primarily in the liver and is considered an indicator of hepatocellular health. ALT level is influenced by disorders such as obesity, insulin resistance, and type-2 diabetes, ailments that wane normal hepatocellular function. Elevated ALT level may result from impaired insulin signaling, since insulin suppresses genes encoding gluconeogenic enzymes, including ALT [40], and/or increased level of TNF-α via inducing hepatic fatty changes [40] characterized by high ALT level. All these disturbances were corrected by treatment with phytol, as presented in the present work

Pioglitazone effect on glucose and lipid homeostasis, acquiesce with previous studies, [40]–[42], as well as its ability to increase adiponectin and to suppress TNF-α, and ALT [43]–[45]. Most of these actions are attributed to the effect of TZDs on PPARγ, as they activate multiple gene cassettes by their robust binding to this nuclear receptor; findings that support our docking results.

As a member of the TZDs, pioglitazone was reported to increase body weight via the activation of PPARγ that causes pre-adipocytes differentiation into mature fat cells and upregulates a number of genes responsible for lipogenesis [32]. However, the present results showed no weight gain in pioglitazone-treated rats; a finding that coincides with earlier studies reporting negative influence of pioglitazone on body weight of high-fat fed rats, with [46], or without [42] STZ. Pioglitazone, unlike rosiglitazone, possesses partial PPARα effect [47] which may clarify its negative influence on body weight.

As PPARs exert their effects by heterodimerizing with RXR, therefore, interaction of either partner with its ligand can regulate the transcriptional activity; this points again to the docking results of both phytanic acid and pioglitazone. In a previous *in vitro* study, co-treatment of cells with ligands for PPAR and RXR resulted in an additive effect [14]. This may explain the results of the current study, where phytol improved almost all altered parameters induced by the current animal model, and its co-administration with pioglitazone (5 mg/kg) exerted better effect, reaching approximately that of the higher pioglitazone dose (10 mg/kg). However, the combined effect was not statistically additive except on decreased body weight, and VFW/BW ratio, both of which have good impact on insulin sensitivity.

In conclusion, phytol has a potential role in the management of insulin resistance and metabolic disorders that accompany diabetes and/or obesity, through activating RXR via its metabolite, and modulating other factors that imply in metabolic disorders. Moreover, molecular docking studies of phytanic acid on the two crystal structures of PPARγ binding protein, and RXRα/PPARγ heterodimer showed good alignment with the experimental findings and verified/confirmed the antidiabetic biological activity of phytol and its active metabolite, phytanic acid. Phytol can be administered with lower doses of TZDs to maintain the full therapeutic action, but with lesser side effects. Therefore, addition of nutraceuticals, at meaningful doses, to antidiabetic agents would have substantial efficacy, and presumably could be used as aids to good glucose tolerance and insulin sensitivity.
